# The Usefulness of Anthropometric Measurements and Indicators in Assessing Muscle Mass in Older Adults

**DOI:** 10.3390/jcm14176067

**Published:** 2025-08-27

**Authors:** Justyna Nowak, Marzena Jabczyk, Paweł Jagielski, Anna Bartosiewicz, Michał Górski, Bartosz Hudzik, Marta Buczkowska, Barbara Zubelewicz-Szkodzińska

**Affiliations:** 1 Department of Cardiovascular Disease Prevention, Department of Metabolic Disease Prevention, Faculty of Public Health in Bytom, Medical University of Silesia, 41-900 Bytom, Poland; bhudzik@sum.edu.pl; 2 Department of Nutrition-Related Disease Prevention, Department of Metabolic Disease Prevention, Faculty of Public Health in Bytom, Medical University of Silesia, 41-900 Bytom, Poland; marzena.jabczyk@gmail.com (M.J.); bzubelewicz-szkodzinska@sum.edu.pl (B.Z.-S.); 3 Department of Nutrition and Drug Research, Faculty of Health Sciences, Institute of Public Health, Jagiellonian University Medical College, 31-008 Krakow, Poland; pawel.jagielski@gmail.com; 4 Faculty of Health Sciences and Psychology, Collegium Medicum, University of Rzeszow, 35-959 Rzeszów, Poland; abartosiewicz@ur.edu.pl; 5 Department of Occupational Medicine and Health, Department of Chronic Diseases and Civilization-Related Hazards, Faculty of Public Health in Bytom, Medical University of Silesia, 41-900 Bytom, Poland; michal.gorski@sum.edu.pl (M.G.); mbuczkowska@sum.edu.pl (M.B.); 6 Third Department of Cardiology, Silesian Center for Heart Disease, Faculty of Medical Sciences in Zabrze, Medical University of Silesia, 41-800 Zabrze, Poland; 7 Department of Endocrinology, District Hospital, 41-945 Piekary Slaskie, Poland

**Keywords:** anthropometric measures, muscle mass, older people, body composition analysis, calf circumference

## Abstract

**Background/Objectives:** Aging is linked to a loss of muscle mass and strength, which increases the risk of falls, disability, and lower quality of life. Identifying individuals at risk of sarcopenia, cachexia, or malnutrition is important. In settings with limited access to advanced diagnostics, simple anthropometric measurements can serve as practical alternatives. This study aimed to evaluate simple, quick, and non-invasive anthropometric measurements as indicators of muscle mass in older adults, based on body composition analysis (BIA). **Methods**: The study included 361 patients admitted to a geriatric ward. Standardized protocols were used to measure anthropometric and body composition parameters, including bioelectrical impedance analysis (BIA). Various body composition indices were calculated, such as body mass index (BMI), body adiposity index (BAI), waist-to-hip ratio (WHR), visceral adiposity index (VAI), fat-free mass index (FFMI), and fat mass index (FMI). **Results**: Strong positive correlations were found between body mass and muscle mass in both women and men. Among anthropometric measures, calf circumference showed the strongest correlation with muscle mass (R = 0.798 in women; R = 0.744 in men, *p* < 0.001). Other indices, including BMI (R = 0.733 in women; R = 0.606 in men, *p* < 0.001), FFMI (R = 0.697 in women; R = 0.721 in men, *p* < 0.001), and FMI (R = 0.600 in women; R = 0.354 in men, *p* < 0.001), were also positively correlated with muscle mass. **Conclusions**: Calf circumference is a simple, quick, and non-invasive measure that can effectively estimate muscle mass in older adults. Although other anthropometric parameters, such as body mass, BMI, FMI, and FFMI, also showed positive correlations with muscle mass, calf circumference appears to be the most practical, as it requires minimal equipment, little time, and limited preparation. This makes it particularly useful for routine assessments in primary care and long-term care settings, where access to advanced diagnostic tools like BIA or DXA is limited. These findings support the use of simple anthropometric measurements as cost-effective and easily accessible alternatives for estimating muscle mass in older adults. Further research is needed to determine appropriate cut-off points for specific populations and sexes.

## 1. Introduction

Throughout life, the human body undergoes various changes due to aging. In older adults, these changes often include an increase in body fat and a decrease in fat-free mass, especially muscle mass. Low muscle mass, often accompanied by reduced muscle strength, is a major concern because it increases the risk of falls, disability, and lower quality of life [1,2,3]. The loss of skeletal muscle can be divided into three main types: starvation-related muscle loss, sarcopenia, and cachexia [2]. Starvation occurs when protein and energy intake are severely insufficient, causing a loss of both fat and fat-free mass [2]. Cachexia refers to severe wasting of fat and fat-free mass, often associated with serious conditions such as cancer or immunodeficiency diseases [2]. Sarcopenia refers to the gradual decline in muscle mass and function in older adults, resulting in reduced mobility, lower quality of life, and an increased risk of fall-related injuries, which may require costly hospital stays and prolonged rehabilitation [1,3].

To assess muscle content in the human body, various tools and methods are available. For screening the risk of malnutrition in older adults, several validated tools can be used, including the Malnutrition Screening Tool (MST), Malnutrition Universal Screening Tool (MUST), Mini Nutritional Assessment–Short Form (MNA-SF), Nutritional Risk Screening 2002 (NRS-2002), and the Subjective Global Assessment (SGA). Additionally, anthropometric measures and indices—such as body mass index (BMI), unintentional weight loss, calf circumference, and mid-arm circumference—can provide valuable insights [4,5].

The European Working Group on Sarcopenia in Older People (EWGSOP2) recommends dual-energy X-ray absorptiometry (DXA) or bioelectrical impedance analysis (BIA) for assessing muscle quantity or quality when identifying sarcopenia [6]. Magnetic resonance imaging (MRI) and computed tomography (CT) are considered gold standards for non-invasive muscle mass assessment. However, their use in primary care is limited due to high costs, lack of portability, and the requirement for specialized personnel. DXA offers a more accessible alternative for non-invasive evaluation of muscle mass, including total body lean tissue and appendicular skeletal muscle mass [6].

Another method for assessing body composition is anthropometry, which involves measuring skinfold thickness, limb circumferences, and body breadths. These measurements can then be applied in validated equations to estimate fat mass, fat-free mass, and muscle mass [7,8]. Anthropometric assessment is a simple and widely used method for evaluating nutritional status in older adults. It can help identify malnutrition, overweight, obesity, muscle loss, fat accumulation, and changes in fat distribution. In addition, anthropometric indicators can predict outcomes in both chronic and acute diseases and guide medical interventions in elderly populations [8].

With the increasing proportion of older adults in the population and the physiological changes associated with aging, early identification of individuals at risk of sarcopenia, cachexia, or malnutrition is essential. These changes have significant consequences for both individual health and public health. Although BIA is a fast, non-invasive, and repeatable method for assessing body composition, its clinical use is often limited by the lack of appropriate equipment in hospitals, clinics, and long-term care facilities. In such settings, anthropometric measurements—which are simple, portable, low-cost, and non-invasive—provide a practical and reliable alternative for evaluating muscle mass and overall nutritional status in older adults. Early detection of health risks using these simple tools allows for the selection of patients who require more detailed diagnostic or comprehensive geriatric assessment. The approach presented in this study has practical and innovative value. It brings advanced methods of nutritional assessment into routine care for older adults, enabling their use not only in clinical research but also in long-term care facilities and other care institutions, where many older individuals reside and access to specialized equipment is limited.

This study aimed to evaluate simple, quick, and non-invasive anthropometric measurements as indicators of muscle mass in older adults, based on body composition analysis.

## 2. Materials and Methods

The study was approved by the Bioethics Committee of the Medical University of Silesia (KNW/0022/KB1/53/14). It followed the ethical guidelines of the Declaration of Helsinki. Written informed consent was obtained from all participants before they joined the study. Data were coded and stored anonymously in a database. Participants were informed that they could withdraw from the study at any time without any consequences.

The study included 422 patients admitted to the geriatric ward of the Piekary Medical Center in Piekary Śląskie, southern Poland, from September 2014 to December 2016, who provided informed consent to participate and had no permanent or temporary disability at the time of the study.

Exclusion criteria included cancer, severe liver disease (e.g., cirrhosis), stage 5 chronic kidney disease (eGFR < 15 mL/min/1.73 m^2^), use of glucocorticoids (oral or inhaled), clinical signs of fluid retention at the time of assessment, mobility limitations, and lack of informed consent. Additionally, individuals with cardiac implantable electronic devices such as pacemakers, implantable cardioverter defibrillators, or cardiac resynchronization therapy devices were excluded from the study due to contraindications for bioelectrical impedance analysis. Based on the exclusion criteria, 41 patients were excluded from the study due to cancer or readmission, 7 patients were using glucocorticosteroids, 3 had severe liver disease, and 6 had cardiac implantable electronic devices. Other patients were excluded for various additional criteria, with some patients meeting multiple exclusion criteria simultaneously. Finally, 361 patients who met all the inclusion criteria were included in the analysis.

### 2.1. Laboratory Measures

Laboratory tests were performed in the morning after an overnight fast. Laboratory tests included the following parameters: albumin (g/L), vitamin D (nmol/L), TSH (µIU/mL), fasting glucose (mmol/L), total cholesterol (mmol/L), LDL cholesterol (mmol/L), HDL cholesterol (mmol/L), non-HDL cholesterol (mmol/L), triglycerides (mmol/L), CRP, eGFR (mL/min/1.73 m^2^), serum creatinine (µmol/L), aspartate aminotransferase (AST, U/L), alanine aminotransferase (ALT, U/L), gamma-glutamyltransferase (GGTP, U/L), total protein (g/L), sodium (mmol/L), and potassium (mmol/L).

### 2.2. Anthropometric Measurements

Anthropometric measurements were conducted personally by the investigator, who had the appropriate expertise. Measurements were performed on the morning of the first day of admission for all patients, following an overnight fast, with participants wearing light outer clothing and no shoes. Standardized procedures and a validated scale were employed for these assessments. Body weight was measured to the nearest 0.01 kg using the Tanita BC 420 S MA Body Composition Analyzer (MDD93/42 EEC certified, Tanita Corp., Tokyo, Japan). Height was measured to the nearest 0.05 cm using the Tanita HR100 Stadiometer (Tanita Corp., Tokyo, Japan). Waist, hip, calf, and upper arm circumferences were measured to the nearest 1 mm using the SECA203 tape measure (Seca, Hamburg, Germany). Skinfold thickness was measured to the nearest 1 mm using the Holtain Caliper (MDD93/42 EEC certified, Holtain Ltd., Crymych, UK).

### 2.3. Anthropometric Indices

Based on the obtained data, individual anthropometric indices were calculated, which are presented in Supplementary Table S1 [9,10,11,12,13,14,15,16,17].

### 2.4. Body Composition Analysis

Body composition analysis was conducted using the Tanita BC 420 S MA Body Composition Analyzer (MDD93/42 certification for medical devices). The measurement system employed tetra-polar bioelectrical impedance analysis. The current frequency during the measurement was 50 kHz, with a current intensity of 90 µA. The measurement range was 150–1200 ohms. The parameters measured included total body composition, such as body fat mass (kg and %), muscle mass, including skeletal muscles, smooth muscles, and the water contained in these muscles (kg), and lean body mass (kg).

### 2.5. Statistical Analysis

Statistical analysis was conducted using STATISTICA 13 PL software (Tulsa, OK, USA). The Shapiro–Wilk test was used to assess the normality of the distribution of variables. For normally distributed data, results are presented as mean ± SD, and for non-normally distributed data, as median with lower and upper quartiles. Normally distributed data were compared using the Student’s *t*-test, while non-normally distributed data were compared with the Mann–Whitney U test. The chi-squared test was used for comparing categorical variables, and the Fisher’s exact test was applied when expected values were small. The Pearson correlation coefficient was used for normally distributed data, and the Spearman rank correlation coefficient was used for non-normally distributed data to assess relationships between variables. A *p*-value of <0.05 was considered statistically significant.

## 3. Results

### Characteristic of the Study Group

No significant difference in age was observed between the groups (*p* = 0.1533). The prevalence of heart failure, coronary artery disease, prior myocardial infarction, atrial fibrillation, stroke, Alzheimer’s disease, dementia, depression, COPD, chronic kidney disease, and diabetes mellitus did not differ significantly between men and women (*p* > 0.05). However, arterial hypertension was significantly more common in women (87.8% vs. 76.4%, *p* = 0.005), whereas men had a higher prevalence of hyperlipidemia (11.4% vs. 5.5%, *p* = 0.043) and anemia (39.0% vs. 23.5%, *p* = 0.002). The comparison of biochemical parameters in the studied group of women and men is presented in Table 1.

Comparison of anthropometric and body composition measurements between the women and men groups is presented in Table 2. Men had significantly higher body mass (75.4 vs. 66.4 kg, *p* < 0.0001), waist circumference (102 vs. 98 cm, *p* = 0.006), and calf circumference (36 vs. 34 cm, *p* < 0.001), while women had a significantly greater hip circumference (104.3 vs. 101.5 cm, *p* = 0.043). Regarding body composition indices, men exhibited significantly higher fat-free mass (55.4 vs. 41.5 kg, *p* < 0.0001) and muscle mass (52.6 vs. 39.4 kg, *p* < 0.0001), while women had significantly higher fat mass (25.2 vs. 19.9 kg, *p* < 0.001) and body fat percentage (37.9% vs. 25.6%, *p* < 0.0001).

The correlation analysis between anthropometric parameters, body composition indices, and muscle mass in both women and men is presented in Table 3.

Body mass showed a strong positive correlation with muscle mass in both sexes (R = 0.834, *p* < 0.001 in women; R = 0.823, *p* < 0.001 in men). The strongest associations among anthropometric measures were observed for calf (R = 0.798 in women; R = 0.744 in men) and hip circumference (R = 0.696; 0.621, *p* < 0.001). BMI and FFMI were significantly correlated with muscle mass in both groups (up to R = 0.733, *p* < 0.001), while FMI and BAI demonstrated stronger associations in women.

## 4. Discussion

The present study has practical significance, as it evaluates the usefulness of simple, quick, and non-invasive anthropometric measurements as an alternative to BIA for assessing muscle mass in older adults, offering a cost-effective diagnostic approach that can be routinely used by healthcare professionals, particularly in resource-limited settings.

In our study, the strongest and most statistically significant correlations were observed between total body mass and muscle mass in both women (R = 0.834) and men (R = 0.823). Despite this strong correlation, it is important to consider the limitations of using body weight to assess muscle mass in older adults. Body weight, while commonly used in nutritional assessments, can be affected by variations in hydration, physiological conditions, and functional impairments that make standing measurements difficult. Furthermore, it may not accurately reflect skeletal muscle mass loss, which can be masked by stable or increased total body weight, as in sarcopenic obesity [8]. In such cases, body circumference measurements—particularly CC—offer a valuable alternative. CC is a simple, rapid, and equipment-free indicator that reflects both lean and fat mass. It is included in the MNA-SF as a substitute for BMI and has been shown to be more effective than mid-arm circumference in identifying malnutrition, except in patients with advanced functional decline [4].

The results of our study demonstrated that among the anthropometric indicators analyzed, CC and BMI showed strong correlations with muscle mass as assessed by BIA. CC, in particular, emerged as a significant and practical indicator, exhibiting a strong correlation with muscle mass in both women (R = 0.798) and men (R = 0.744). These indicators are among the simplest and fastest to obtain, requiring only basic equipment such as a scale and a measuring tape. Additionally, the formulas used to calculate indices like BMI are straightforward and easy to apply. For these reasons, such parameters are especially valuable for rapid, screening-level assessments of muscle mass and, by extension, nutritional status—particularly in environments where older adults reside and where access to specialized diagnostic equipment is limited. Importantly, these assessments can be conducted by general medical staff without the need for advanced training, making them highly practical for routine clinical use and in long-term care settings. In our study a relatively high correlation coefficient was also observed for the FFMI, with R = 0.721 for men and R = 0.697 for women. However, calculating this index requires data on body fat content, which is typically obtained through methods such as BIA. Similarly, the AVI demonstrated a moderately strong correlation with muscle mass (R = 0.625 for men and R = 0.602 for women). However, in addition to anthropometric measurements, calculating AVI requires biochemical data, including triglyceride and HDL cholesterol levels. As a result, its utility in rapid screening contexts is limited. The practical application of this index is therefore constrained, particularly in environments lacking access to laboratory testing or advanced diagnostic tools. These findings reinforce the value of simple anthropometric measurements—particularly calf circumference, when adjusted for sex—as viable alternatives to BIA for estimating muscle mass in older adults, especially in healthcare settings with limited diagnostic resources.

According to the European Working Group on Sarcopenia in Older People (EWGSOP2), CC has been shown to be a reliable predictor of physical performance and survival in older adults, with a proposed cut-off point of <31 cm [6]. In resource-limited settings where advanced methods such as DXA or BIA are not available, the Asian Working Group for Sarcopenia (AWGS) 2019 recommends using CC as a simple, non-invasive, and cost-effective surrogate for muscle mass assessment. The group suggests threshold values of <34 cm for men and <33 cm for women [6]. This anthropometric measure has demonstrated strong potential for sarcopenia screening and has been associated with an increased risk of mortality when below the recommended thresholds [18].

A slight positive correlation between muscle mass measured by BIA and calf circumference (CC) (r = 0.312, *p* < 0.001) was reported in 105 adults over 60 from a Mexican geriatric clinic [19]. The relatively low correlation may reflect the lack of sex-specific analysis, an approach applied in the present study, which revealed significant differences between men and women. Kiss et al. [20], in a study of 305 patients admitted to Swiss acute and rehabilitation geriatric wards, found a positive association between CC and the appendicular skeletal muscle mass index (ASMI). Optimized CC cut-offs for low ASMI were <31.5 cm for women and <33.5 cm for men, supporting CC as a practical indicator of low muscle mass when imaging-based assessment is unavailable. In a study of Brazilian women aged ≥65 years, muscle mass was significantly correlated with body weight, arm, waist, hip, and calf circumferences, and regression analysis identified body weight and CC as independent predictors of muscle mass (r = 0.81, r^2^ = 0.65, F = 55.7) [21]. In another elderly Brazilian population, the most accurate CC cut-offs for reduced muscle mass were 34 cm for men and 33 cm for women [22]. In older Japanese adults, CC was strongly correlated with DXA-measured appendicular skeletal muscle mass (ASM) and skeletal muscle index (SMI) (r = 0.73–0.81 in men and women). Receiver operating characteristic (ROC) analysis identified optimal CC cut-offs for sarcopenia of 34 cm for men and 33 cm for women [23]. In community-dwelling Thai adults aged ≥ 60, CC was strongly correlated with SMI (r = 0.75, *p* < 0.001). Optimal CC cut-offs for low muscle mass were 34.0 cm for women and 33.4 cm for men, demonstrating good diagnostic accuracy and supporting CC as a practical screening tool in community and primary care settings [24]. In a separate study involving an older Thai population, optimal CC cut-offs for low muscle mass were <33 cm for women and <34 cm for men. In adults aged ≥75, these cut-offs remained unchanged but showed improved diagnostic performance [25].

Numerous studies have highlighted the usefulness of CC in assessing muscle mass in older adults, as well as its indirect role in evaluating nutritional status in settings lacking access to specialized equipment. In the present study, specific cut-off values for calf circumference were not established, as explained in the Study Limitations section. According to the EWGSOP2 guidelines, the recommended threshold is <31 cm. However, it is important to note that studies conducted in Asia [18] have demonstrated sex-specific differences in optimal cut-off values. Therefore, further research in European populations is warranted to define sex-specific thresholds for both women and men.

Reduced muscle mass is a key diagnostic criterion in the GLIM consensus. However, there is currently no universally accepted method for its assessment, particularly in clinical settings. GLIM recommends the use of DXA or other validated techniques such as BIA, ultrasound, CT, or MRI. Nonetheless, the global availability of these technologies remains limited. As a result, physical examinations and anthropometric measurements—such as calf or mid-arm muscle circumference—are proposed as practical alternatives. These recommendations may continue to evolve alongside advances in portable and more affordable body composition assessment technologies [26].

In the context of an aging population and the increasing prevalence of sarcopenia and malnutrition, there is an urgent need to implement simple, accessible, and effective methods for assessing muscle mass and nutritional status. The findings of the present study confirm that CC is a practical and reliable indicator of muscle mass in older adults, particularly in resource-limited settings. This measurement demonstrates a strong correlation with muscle mass assessed via BIA and shows high sensitivity and specificity in identifying individuals with low muscle mass—results that are consistent with findings from numerous international studies, including those conducted in Japan, Brazil, Switzerland, and Thailand.

Clinically, our results indicate that calf circumference can serve as a practical first-line screening tool in everyday medical practice. Its simplicity and low cost make it feasible for use in primary care and long-term care settings, enabling early identification of older adults at risk of muscle loss or malnutrition and supporting timely referral for further assessment or intervention.

As a simple, quick, and non-invasive method, CC can serve as an effective alternative to more advanced techniques such as DXA, MRI, or BIA, which are often unavailable in primary healthcare or long-term care facilities. In line with the recommendations of the GLIM and sarcopenia working groups (EWGSOP2, AWGS), CC measurement can be used as a phenotypic criterion in diagnosing malnutrition and sarcopenia. However, the lack of clearly defined cut-off values for the European population, particularly with regard to sex-specific differences, highlights the need for further research in this area.

### Study Limitations

Despite the authors’ best efforts in designing and conducting the study, several important limitations should be acknowledged. The most significant of these is that the body composition analysis device used in the study only provided an estimate of total body mass, without the ability to distinguish skeletal muscle mass. As a result, it was not possible to apply the EWGSOP2 criteria to assess normal or reduced muscle mass in the study group, which in turn prevented the determination of specific cut-off points for the analyzed parameters in relation to low muscle mass. While the authors are aware that formulas exist to estimate skeletal muscle mass based on BIA-derived data, the use of such methods could introduce a higher risk of error, potentially compromising the reliability of the findings. Another limitation of this study is that some statistically significant results (e.g., hip circumference, *p* = 0.0433) may not reflect meaningful effects, as effect sizes were not calculated.

## 5. Conclusions

The results confirm that calf circumference (CC) is a simple, quick, and non-invasive measure that can effectively estimate muscle mass in older adults. Although other anthropometric parameters, such as body mass, BMI, FMI, and FFMI, also showed positive correlations with muscle mass, calf circumference appears to be the most practical, as it requires minimal equipment, little time, and limited preparation. This makes it particularly useful for routine assessments in primary care and long-term care settings, where access to advanced diagnostic tools like BIA or DXA is limited. These findings support the use of simple anthropometric measurements as cost-effective and easily accessible alternatives for estimating muscle mass in older adults. Further research is needed to determine appropriate cut-off points for specific populations and sexes.

## Figures and Tables

**Table 1:** Baseline clinical and laboratory characteristics.

	n Women	Women Group (n = 238)	n Men	Men Group (n = 123)	*p*
Age (years)	238	78 (72–83)	123	77 (70–83)	0.153 *
Heart failure n (%)	238	73 (30.7)	123	37 (30.1)	0.908 **
Coronary artery disease n (%)	238	85 (35.7)	123	45 (36.6)	0.870 **
Prior myocardial infarction n (%)	238	13 (5.5)	123	12 (9.8)	0.128 **
Atrial fibrillation n (%)	238	39 (16.4)	123	28 (22.8)	0.139 **
Arterial hypertension n (%)	238	209 (87.8)	123	94 (76.4)	0.005 **
Stroke n (%)	238	22 (9.2)	123	11 (8.9)	0.925 **
Alzheimer’s disease n (%)	238	25 (10.5)	123	7 (5.7)	0.171 **
Dementia n (%)	238	34 (14.3)	123	13 (10.6)	0.320 **
Depression n (%)	238	18 (7.6)	123	7 (5.7)	0.663 ****
Chronic obstructive pulmonary disease n (%)	238	10 (4.2)	123	11 (8.9)	0.068 **
Chronic kidney disease (stage 1–4) n (%)	238	43 (18.1)	123	15 (12.2)	0.149 **
Diabetes mellitus n (%)	238	88 (37.0)	123	53 (43.1)	0.259 **
Mixed hyperlipidemia n (%)	238	13 (5.5)	123	14 (11.4)	0.043 **
Hypercholesterolemia n (%)	238	73 (30.7)	123	15 (12.2)	<0.001 **
Hypertriglycerides n (%)	238	3 (1.3)	123	0 (0.0)	0.554 ****
Hypothyroidism n (%)	238	43 (18.1)	123	8 (6.5)	0.003 **
Osteoporosis n (%)	238	22 (9.2)	123	4 (3.3)	0.051 ****
Anemia n (%)	238	56 (23.5)	123	48 (39.0)	0.002 **
Albumin (g/L)	131	36 (34–38)	58	36 (34–38)	0.871 *
Vitamin D (nmol/L)	190	33.9 (25.9–46.2)	80	40.2 (30.3–49.7)	0.031 *
TSH (uIU/mL)	230	1.3 (0.7–1.9)	114	1.3 (0.9–1.9)	0.605 *
Fasting glucose (mmol/L)	237	5.4 (4.9–6.3)	123	5.6 (5.1–6.4)	0.316 *
Total cholesterol (mmol/L)	234	4.8 (4.0–5.8)	119	4.3 (3.5–5.2)	0.005 *
LDL cholesterol (mmol/L)	235	2.8 (2.1–3.5)	119	2.4 (1.9–3.2)	0.065 *
HDL cholesterol (mmol/L)	235	1.5 (1.2–1.8)	119	1.1 (0.9–1.4)	>0.0001 *
Non-HDL cholesterol (mmol/L)	233	3.3 (2.6–4.1)	119	3.1 (2.5–3.9)	0.295 *
Triglycerides (mmol/L)	235	1.2 (0.9–1.5)	117	1.3 (1.0–1.8)	0.015 *
CRP	234	1.6 (0.7–4.0)	118	2.9 (1.0–7.3)	0.001 *
eGFR (mL/min/1.73 m^2^)	201	70.8 (±21.1)	97	76.8 (±23.4)	0.017 ***
Serum creatinine (µmol/L)	236	0.8 (0.7–0.9)	123	0.9 (0.8–1.2)	<0.0001 *
Aspartate aminotransferase AST (U/L)	238	18 (15.0–23.0)	123	18 (15–26)	0.507 *
Alanine aminotransferase ALT (U/L)	238	16 (12.0–22.0)	123	17 (12–25)	0.202 *
Gamma-glutamyltransferase GGTP (U/L)	232	20 (14–34)	118	27.5 (19–46)	<0.0001 *
Total protein (g/L)	118	64.9 (61.5–68)	66	64.7 (59–67.4)	0.428 *
Sodium (mmol/L)	238	141 (139–143)	122	140 (139–142)	0.097 *
Potassium (mmol/L)	238	4.4 (4.1–4.7)	122	4.3 (4.1–4.7)	0.452 *

* U Mann–Whitney test; ** Chi-squared test; *** Student’s *t*-test; **** Fisher’s test.

**Table 2:** Comparison of anthropometric and body composition measurements between the women and men groups.

	n	Women Group	n	Men Group	*p*
Body mass (kg)	238	66.4 (56.3–75.3)	123	75.4 (68–88.8)	<0.0001 *
Height (cm)	238	153.3 (149.5–158)	123	167 (162–171.5)	<0.0001 *
Waist circumference (cm)	238	98 (90–107)	123	102 (95–110)	0.006 *
Hip circumference (cm)	238	104.3 (97–113)	123	101.5 (97–107)	0.043 *
Arm circumference (cm)	238	28.7 (±4.2)	123	29.2 (±3.9)	0.317 **
Calf circumference (cm)	238	34 (32–37)	123	36 (33.5–38)	<0.001 *
MAMC (cm)	235	21.9 (19.8–23.7)	117	24.7 (22.5–26.3)	0.031 *
BMI (kg/m^2^)	238	27.7 (24.6–31.5)	123	27.8 (±4.4–27.5)	0.647 *
BAI (%)	238	36.9 (32.7–41.7)	123	29.4 (26.7–32.1)	<0.0001 *
VAI	235	1.7 (1.1–2.7)	117	1.6 (1.2–2.7)	0.615 *
WHR	238	0.9 (±0.07)	123	1.0 (±0.06)	<0.0001 **
WHtR	238	0.65 (±0.09)	123	0.62 (±0.07)	0.002 **
FFMI (kg/m^2^)	238	17.3 (16.1–19.0)	123	20.2 (18.7–21.4)	<0.0001 *
FMI (kg/m^2^)	238	10.4 (8.1–13.3)	123	7.4 (5.8–8.9)	<0.0001 *
BRI	238	6.5 (5.1–7.9)	123	5.8 (4.7–6.8)	0.002 *
ABSI	238	0.09 (±0.01)	123	0.09 (±0.0)	0.610 **
AVI	238	19.2 (16.3–22.9)	123	20.8 (18.1–24.2)	0.009 *
Body fat (kg)	238	25.2 (18.4–32.2)	123	19.9 (15.5–26.0)	<0.001 *
Body fat (%)	238	37.9 (32.5–43.0)	123	25.6 (22.4–31.6)	<0.0001 *
Muscle mass (kg)	238	39.4 (36.3–42.1)	123	52.6 (49–57.5)	<0.0001 *
Free fat mass (kg)	238	41.5 (38.3–44.4)	123	55.4 (51.6–60.5)	<0.0001 *

* U Mann–Whitney test; ** Student’s *t*-test.

**Table 3:** Correlation between anthropometric parameters, indices, and muscle mass in the women and men groups.

		Women Group		Men Group	
		R	*p*-Value	R	*p*-Value
Body mass (kg)	Muscle mass [kg]	0.834	<0.001	0.823	<0.001
Height (cm)		0.381	<0.001	0.607	<0.001
Waist circumference (cm)		0.597	<0.001	0.626	<0.001
Hip circumference (cm)		0.696	<0.001	0.621	<0.001
Arm circumference (cm)		0.651	<0.001	0.560	<0.001
Calf circumference (cm)		0.798	<0.001	0.744	<0.001
MAMC (cm)		0.548	<0.001	0.363	<0.001
BMI (kg/m^2^)		0.733	<0.001	0.606	<0.001
BAI (%)		0.443	<0.001	0.069	0.450
VAI		0.130	0.047	0.232	0.012
WHR		0.021	0.745	0.339	<0.001
WHtR		0.456	<0.001	0.327	<0.001
FFMI (kg/m^2^)		0.697	<0.001	0.721	<0.001
FMI (kg/m^2^)		0.600	<0.001	0.354	<0.001
BRI		0.46	<0.001	0.327	<0.001
ABSI		−0.234	<0.001	−0.225	0.012
AVI		0.602	<0.001	0.625	<0.001
Body fat (%)		0.440	<0.001	0.185	0.041
Body fat (kg)		0.653	<0.001	0.483	<0.001

## Data Availability

The data are available from the corresponding author upon reasonable request.

## Supplementary Materials

The following supporting information can be downloaded at: https://www.mdpi.com/article/10.3390/jcm14176067/s1, Table S1: Anthropometric and Body Composition Indices.

## Abbreviations

BIA	Bioelectrical Impedance Analysis
MST	Malnutrition Screening Tool
MUST	Malnutrition Universal Screening Tool
MNA-SF	Mini Nutritional Assessment–Short Form
NRS-2002	Nutritional Risk Screening 2002
SGA	Subjective Global Assessment
BMI	Body Mass Index
EWGSOP2	The European Working Group on Sarcopenia in Older People
DXA	Dual-Energy X-ray Absorptiometry
MR	Magnetic Resonance
CT	Computed Tomography
ASM	Skeletal Muscle Mass
BAI	Body Adiposity Index
WHR	Waist–Hip Ratio
WHtR	Waist–Height Ratio
VAI	Visceral Adiposity Index
BRI	Body Roundness Index
ABSI	A Body Shape Index
AVI	Abdominal Volume Index
FFMI	Fat-Free Mass Index
FMI	Fat Mass Index
MAMC	Mid-Arm Muscle Circumference
TS	Triceps Skinfold
