# Supplementary material for: The Usefulness of Anthropometric Measurements and Indicators in Assessing Muscle Mass in Older Adults
Source: J Clin Med. 2025 Aug 27;14(17):6067. doi: 10.3390/jcm14176067 (PMC12429639; doi:10.3390/jcm14176067)
Supplement: Supplementary file 1 [file jcm-14-06067-s001.zip › jcm-3821481-supplementary.pdf]

## Supplementary Table S1

Table 1. Anthropometric and Body Composition Indices

| Name of Index | Formula | References |
|---|---|---|
| Body Mass Index (BMI) | BMI= weight [kg] / height $[m]^2$ | [9] |
| Body Adiposity Index (BAI) | BAI= hip circumference [cm] / height $[m]^{1.5}$- 18 | [10] |
| Waist-hip ratio (WHR) | WHR= waist circumference [cm]/hip circumference [cm] | [11] |
| Waist-height ratio (WHtR) | WHtR= waist circumference [cm]/height [cm] | [11] |
| Visceral Adiposity Index (VAI) | Men: VAI= [waist circumference [cm]/(39.68 + (1.88 x BMI))] x (triglyceride concentration [mmol/L] / 1.03) x (1.31/HDL concentration [mmol/L]) | [12] |
| | Women: VAI= [waist circumference [cm]/(36.58 + (1.89 x BMI))] x (triglyceride concentration [mmol/L] / 0.81) x (1.52/HDL concentration [mmol/L]) | |
| Body Roundness Index (BRI) | BRI=365.2 − 365.5 × √(1 − ((WC/2π)2)/[(0.5 × height)]$^2$ | [13] |
| A Body Shape Index (ABSI) | ABSI= WC[m]/[$(BMI)^{2/3}$) x (height $[m])^{1/2}$) | [14] |
| Abdominal Volume Index (AVI) | AVI= [2 x (waist circumference in cm) 2+0.7x (waist circumference in cm – hip circumference in cm)$^2$]/ 1000 | [15] |
| Fat free mass index (FFMI) | FFMI=free fat mass [kg] / height $[m]^2$ | [16] |
| Fat mass index (FMI) | FMI=fat mass [kg] / / height $[m]^2$ | [16] |
| The Mid-Arm Muscle Circumference (MAMC) | MAMC= mid-arm circumference [cm] − 0.3142 × triceps skinfold (TS) thickness [mm] | [17] |
